# Transtibial ACL reconstruction produces higher sagittal inclination angles than the native ACL: A systematic review and meta‐analysis of MRI‐based measurements in 2047 knees

**DOI:** 10.1002/jeo2.70840

**Published:** 2026-07-06

**Authors:** Ying Ren Mok, Chen Wang, Francis Jia Yi Fong, Ming Wang, Yee Han Dave Lee, James Hui, Stefano Zaffagnini

**Affiliations:** ^1^ Department of Orthopaedic Surgery National University Hospital Singapore Singapore; ^2^ Department of Orthopaedic Surgery McGovern Medical School at UT Health Houston Texas USA; ^3^ Clinica Ortopedica e Traumatologica II IRCCS Istituto Ortopedico Rizzoli Bologna Italy; ^4^ Dipartimento di Scienze Biomediche e Neuromotorie (DIBINEM) University of Bologna Bologna Italy

**Keywords:** anatomic reconstruction, coronal inclination angle, graft inclination angle, graft orientation, over‐the‐top technique, sagittal inclination angle

## Abstract

**Purpose:**

The aim of this systematic review and meta‐analysis was twofold: (1) to synthesize magnetic resonance imaging (MRI)‐based sagittal inclination angle (SIA) and coronal inclination angle (CIA) of the native anterior cruciate ligament (ACL) using standardized, tibial‐axis–referenced measurements and (2) to compare inclination angles achieved by contemporary ACL reconstruction techniques relative to native ACL anatomy.

**Methods:**

A systematic search of MEDLINE, Embase and Cochrane CENTRAL was conducted in accordance with Preferred Reporting Items for Systematic Reviews and Meta‐analysis (PRISMA) guidelines. The review was registered on Open Science Framework (OSF); registration number 10.17605/OSF.IO/K8HWRDEDXX. Studies reporting SIA and/or CIA of the native ACL or reconstructed ACL graft measured on MRI using tibial‐axis–referenced techniques on true sagittal (for SIA) and true coronal (for CIA) imaging planes were included. Studies using alternative reference frames, oblique imaging, non‐MRI modalities, cadaveric or animal models, double‐bundle reconstruction or predominantly skeletally immature populations were excluded. Random‐effects meta‐analyses calculated pooled mean inclination angles with 95% confidence intervals (CIs).

**Results:**

Twenty‐one studies comprising 2047 knees met the inclusion criteria. The pooled mean native ACL sagittal inclination angle was 50.4° (95% CI 49.0°–52.0°), whereas the pooled native coronal inclination angle was higher and more variable at 67.5° (95% CI 63.3°–71.8°). Anteromedial portal and outside‐in techniques achieved sagittal and coronal graft orientations comparable to native ACL values. In contrast, transtibial reconstruction demonstrated significantly higher sagittal inclination angles. No significant differences in coronal inclination were observed across techniques.

**Conclusion:**

Sagittal graft orientation differs by technique, whereas coronal inclination shows substantial overlap, providing an anatomy‐referenced framework for interpreting MRI‐based graft alignment.

**Level of Evidence:**

Level IV, systematic review and meta‐analysis of Level III and IV studies.

**Abbreviations**
CIA: coronal inclination angle
SIA: sagittal inclination angle

## INTRODUCTION

Anterior cruciate ligament (ACL) reconstruction is among the most commonly performed procedures in sports medicine, with incidence rising alongside participation in pivoting and cutting sports. Despite decades of technical refinement, graft failure and suboptimal outcomes persist, with femoral tunnel malposition [45, 52] consistently identified as the leading technical cause. Large registry and revision cohort studies, including data from the Multicenter ACL Revision Study (MARS) cohort [36], indicate that femoral malposition accounts for the majority of technical failures and approximately 80% of revision cases [40], often necessitating creation of a new femoral tunnel [52].

In response, surgical techniques have evolved from transtibial drilling toward anteromedial portal [30, 50] and outside‐in approaches that permit independent femoral tunnel placement; however, malposition has not been uniformly eliminated [9, 26, 33], and femoral tunnel–independent strategies such as over‐the‐top reconstruction [35, 57] have also been described as alternatives.

The concept of ‘anatomic’ ACL reconstruction has therefore gained widespread acceptance, with increasing emphasis on reproducing native ligament orientation and function [6]. However, anatomic reconstruction remains variably defined [55], and objective comparison of graft placement across techniques remains challenging. As a result, magnetic resonance imaging (MRI)–based surrogate measures of graft orientation—most notably sagittal and coronal inclination angles [15]—have been widely adopted.

The biomechanical relevance of MRI‐derived sagittal inclination angle (SIA) was established by Illingworth and colleagues [25], who demonstrated a strong association between increasing SIA and anterior femoral tunnel placement on three‐dimensional computed tomography, thereby linking MRI‐based inclination measures to true tunnel anatomy. Furthermore, these angles have shown strong correlation with computer‐assisted navigation–based assessments of graft orientation [58], supporting their use as meaningful descriptors of graft orientation. The coronal inclination angle (CIA), measured between the medial border of the ACL and the tibial plateau on sequential coronal MRI slices, is a complementary but less extensively validated MRI descriptor of graft orientation. Although fewer studies have evaluated its clinical or biomechanical correlates compared with SIA, CIA is routinely reported in contemporary imaging studies and is included alongside SIA in current consensus measurement frameworks.

Accordingly, the purpose of this systematic review and meta‐analysis was twofold: (1) to quantify sagittal and CIAs of the native ACL using MRI‐based, tibial‐referenced measurements and (2) to compare contemporary single‐bundle reconstruction techniques (transtibial, anteromedial portal with rigid or flexible reamers and outside‐in/retrograde drilling) against this anatomic reference. It was hypothesized that anteromedial portal and outside‐in/retrograde drilling techniques would reproduce SIAs and CIAs comparable to the native ACL, whereas transtibial reconstruction would demonstrate systematically higher SIAs.

## METHODS

### Search strategy

The present systematic review was conducted in accordance with the Preferred Reporting Items for Systematic Reviews and Meta‐analysis (PRISMA) statement. An electronic database search of PubMed, Embase, Scopus and Web of Science was performed using the keywords: Anterior cruciate ligament, graft sagittal and coronal orientation or inclination angles and MRI. The detailed search strategy is shown in Supporting Information S1: Table 1.

### Data assessment, inclusion and exclusion criteria

Abstracts were independently screened by two reviewers (Mok Ying Ren and Francis Fong) to remove duplicates and selected based on the predetermined inclusion criteria (Table 1). Native ACL data were drawn from studies imaging either (a) the contralateral uninjured knee of patients undergoing unilateral ACL reconstruction or (b) the index knee of patients diagnosed with conditions other than ACL injury. The full texts of the remaining studies were further analysed. Reference lists of included articles were hand searched to identify further studies for analysis. Any discrepancies were resolved by achieving a consensus with a third author (Wang Chen).

**Table 1:** Inclusion and exclusion criteria.

Inclusion	Exclusion
− Clinical studies utilizing transtibial, antero‐medial portal (flexible and/or rigid), outside‐in or over‐the‐top techniques. − Clinical studies that reported sagittal and/or coronal inclination angles of the native and/or reconstructed ACL graft measured on MRI using a tibial axis–referenced measurement technique. − Studies employing true sagittal MRI planes. − Studies whose cohort includes predominantly skeletally mature patients. − For native ACL data: Patients with imaging confirmation of an intact native ACL, from either (a) the contralateral uninjured knee of patients undergoing unilateral ACL reconstruction or (b) the index knee diagnosed with other conditions other than ACL injury.	− Biomechanical studies − Cadaveric studies − In vitro studies − Animal studies − Review articles, case reports, conference papers and letters which do not contain original data − Non‐English language articles − Studies using alternative reference frames (e.g., tibial plateau–based sagittal references), oblique sagittal imaging planes [22], non‐MRI imaging modalities or non‐comparable measurement techniques [9, 23] − Double‐bundle ACL reconstruction [8] studies. − Native ACL data drawn from patients with prior ipsilateral knee surgery

Abbreviations: ACL, anterior cruciate ligament; MRI, magnetic resonance imaging.

### Data collection

**Figure 1:**
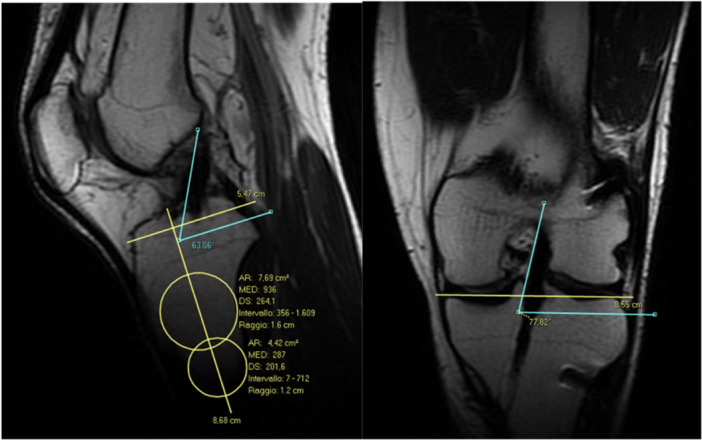
Tibia axis‐based measurement of sagittal inclination angle. Coronal inclination angle measured with reference to the tibial plateau.

Data from included studies were extracted independently by two authors using a standardized protocol and reporting form. The extracted data included the following: study characteristics (year of study, study design, patient demographics), radiological outcomes (ACL graft SIA and/or CIA), reconstruction techniques and graft type. When means and standard deviation data were unavailable, conversions of data were performed using previously established models by Hozo et al. [24] and Wan et al. [54]. The following definitions were applied:

1. Sagittal inclination angle (SIA) was defined as the angle between the longitudinal axis of the ACL (or graft) and a line perpendicular to the tibial long axis on the sagittal image that best visualized the ligament, consistent with the method described by Mellado et al. [37] and Ahn et al. [1]. The tibial axis was determined using validated diaphyseal‐based circular or linear techniques and corresponds to reference standards adopted in contemporary consensus recommendations [4, 51] (Figure 1).
2. Coronal inclination angle (CIA) assessment was performed on coronal MRI sequences. As the entire ACL is rarely visualised.
3. Based on a single coronal slice, the medial margin of the ligament was followed across sequential images. The medial edge of the femoral ACL attachment was first identified, after which images were scrolled in the anterior–posterior direction to locate the medial edge of the tibial ACL attachment. A line connecting these two points, representing the medial border of the ACL, was then compared with the articular surface of the tibial plateau to determine the CIA [1] (Figure 1).

### Quality assessment

To evaluate bias and quality in this single‐arm meta‐analysis, a risk of bias assessment approach was adopted based on the method designed specifically for single‐arm studies [23] (Supporting Information S1: Table 2). Quality assessment was performed independently by two reviewers (Mok Ying Ren and Francis Fong), with disagreements resolved by consensus. The Newcastle–Ottawa Quality Assessment Scale was used for the assessment of the quality of non‐randomized studies in meta‐analyses [46].

### Statistical analysis

A single‐arm meta‐analysis was performed using RStudio (Version 213, 2024.09.01) for all extracted continuous data. The *metamean* function was then executed using mean values, sample sizes and standard deviations.

Following the analysis, the overall effect size results provided the mean estimate and 95% confidence interval (CI). Subgroup analyses displayed the mean estimates and heterogeneity metrics for each group, with subgroup differences assessed using the *Q* test. Subgroup comparisons were performed between the native ACL group and each reconstruction technique subgroup (outside‐in/retrograde, anteromedial portal–flexible, anteromedial portal–rigid and transtibial) for both SIA and CIA. The threshold for statistical significance was set at *p* < 0.05. Given the anticipated heterogeneity in patient populations, MRI acquisition protocols and surgical technique variants across the included studies, a random‐effects model was selected a priori, as it explicitly accounts for between‐study variability and does not rely on the assumption of a single underlying true effect. Pooled means were calculated using the inverse‐variance method via the *metamean* function in R, with between‐study variance (*τ* ^2^) estimated using restricted maximum likelihood (REML). REML was preferred over the DerSimonian–Laird method as it produces less biased estimates of *τ* ^2^ when the number of studies is small and between‐study heterogeneity is high, both of which applied to several subgroups in this analysis. It is acknowledged that under conditions of very high heterogeneity, the inverse‐variance weights become dominated by *τ* ^2^ and converge toward near‐equality across studies, such that the pooled estimate approximates an unweighted average of study‐level means [53]; this consequence is addressed in the Discussion limitations.

Heterogeneity within subgroups and between two subgroups was evaluated using the *I* ^2^ value. Heterogeneity was classified as minimal (*I* ^2^ 0%–30%), moderate (*I* ^2^ 30%–60%) or significant (*I* ^2^ > 60%), per established thresholds [21].

## RESULTS

**Figure 2:**
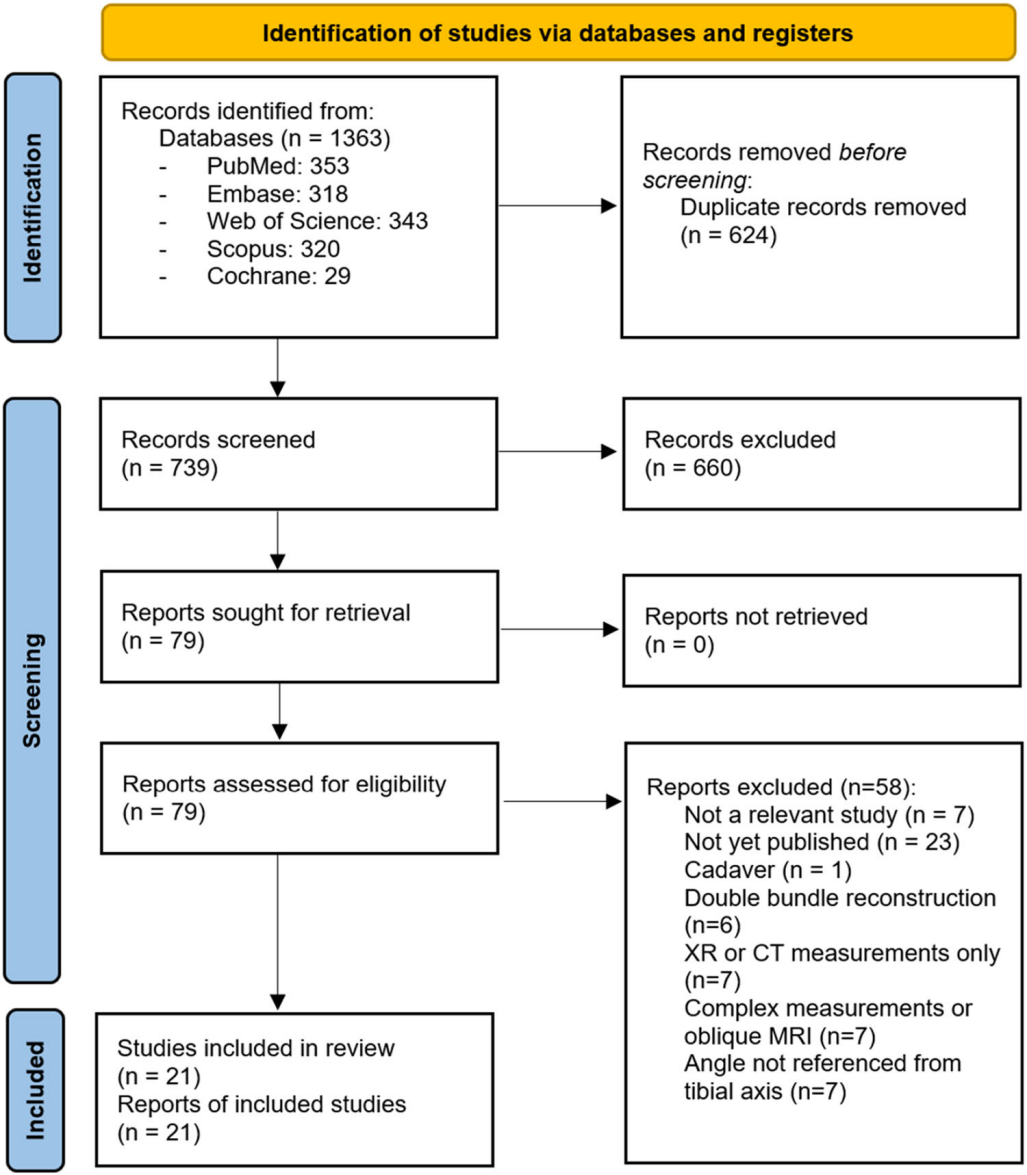
PRISMA flow diagram. CT, computed tomography; MRI, magnetic resonance imaging; PRISMA, Preferred Reporting Items for Systematic Reviews and Meta‐analysis; XR, X‐ray.

A systematic search of the literature using the predefined search strategy yielded a total of 1363 articles, with 739 remaining after removal of duplicates. Six hundred and sixty articles were excluded based on title and abstract review. The remaining 79 articles underwent full‐text review, of which 21 articles were subsequently included in the meta‐analysis (Figure 2).

All 21 included studies were of good quality according to the Newcastle‐Ottawa scale, with a range of 7–9 points (Supporting Information S1: Table 3). Seventeen studies were retrospective cohort studies, one was a randomized controlled trial, and the remaining three were prospective cohort studies, comprising a total of 2047 knees, including 741 native ACLs and 1306 reconstructed ACLs. Six studies originated from Europe, four each from the United States and Korea, two each from China and Japan, while one study each was conducted in Argentina, Turkey and India.

The mean age of participants in the native ACL group was 31.3 ± 6.2 years. Among the surgical technique subgroups, three studies evaluating the outside‐in/retrograde drilling technique reported a pooled mean age of 29.1 ± 2.0 years. Two studies assessing anteromedial portal drilling with a flexible reamer demonstrated a mean age of 30.9 ± 3.5 years. Thirteen studies investigating anteromedial portal drilling with a rigid reamer reported a mean age of 30.2 ± 3.2 years, while 12 studies evaluating the transtibial technique reported a mean age of 30.9 ± 5.4 years. Sex distribution was reported in sixteen studies, comprising 1100 male and 354 female participants.

Reconstruction techniques included transtibial, anteromedial portal drilling (rigid and flexible), outside‐in and retrograde drilling approaches. Hamstring tendon autografts were most used (*n* = 11 studies), followed by bone–patellar tendon–bone autografts (*n* = 4 studies), quadriceps tendon autografts (*n* = 2 studies) and select allografts (*n* = 4 studies). Further details of the demographic data of the papers included are shown in Table 2 and in Supporting Information S1: Table 4.

**Table 2:** Demographic details of studies included.

Study population	Total number of studies reporting value of interest	Graft types	Study design	Total number of knees included	Mean age	Total number of males	Mean SIA (°)	Mean CIA (°)
Native ACL	14	NA	11 RCS; 3 PCS	741	31.3 ± 6.2	304	50.5 ± 3.2	67.6 ± 3.8
Outside‐in/retrograde drilling	3	3 HS	2 RCS; 1 PCS	233	29.1 ± 2.0	191	52.6 ± 5.2	70.1 ± 7.4
Anteromedial portal drilling (flexible)	2	2 HS	1 RCS; 1 PCS	78	30.9 ± 3.5	50	51.4 ± 4.8	69.2 ± 4.6
Anteromedial portal drilling (rigid)	13	7 HS; 3 BPTB; 1 QT; 2 Others	10 RCS; 2 PCS; 1 RCT	503	30.2 ± 3.2	189	53.2 ± 4.6	60.8 ± 4.7
Transtibial drilling	12	6 HS 1 HS and BPTB 2 HS and TAA 1 BPTB 1 QT 1 Others	9 RCS; 3 PCS	492	30.9 ± 5.4	366	61.2 ± 5.8	70.6 ± 5.0

Abbreviations: ACL, anterior cruciate ligament; BPTB, bone–patellar tendon–bone; CIA, coronal inclination angle; HS, hamstring tendon autografts; NA, not applicable; PCS, prospective cohort study; QT, quadriceps tendon; RCS, retrospective cohort study; RCT, randomized controlled trial; SIA, sagittal inclination angle; TAA, tibialis tendon allograft.

### SIA

#### Native ACL

Fourteen studies [2, 3, 5, 7, 8, 10, 16, 18, 27, 31, 42, 49] reported SIAs of the native ACL using tibial‐axis–based MRI measurements. The pooled mean native ACL SIA was 50.4° (95% CI 49.0°–52.0°), with significant heterogeneity observed across studies (*I^2^* ≈ 97.2%, *p* < 0.001). Study‐level mean SIA values ranged from 44.5° to 58.7° across the 14 included native ACL studies. Despite this heterogeneity, study‐level means clustered within a relatively narrow range centred around 50°.

### Outside‐in/Retrograde drilling

**Figure 3:**
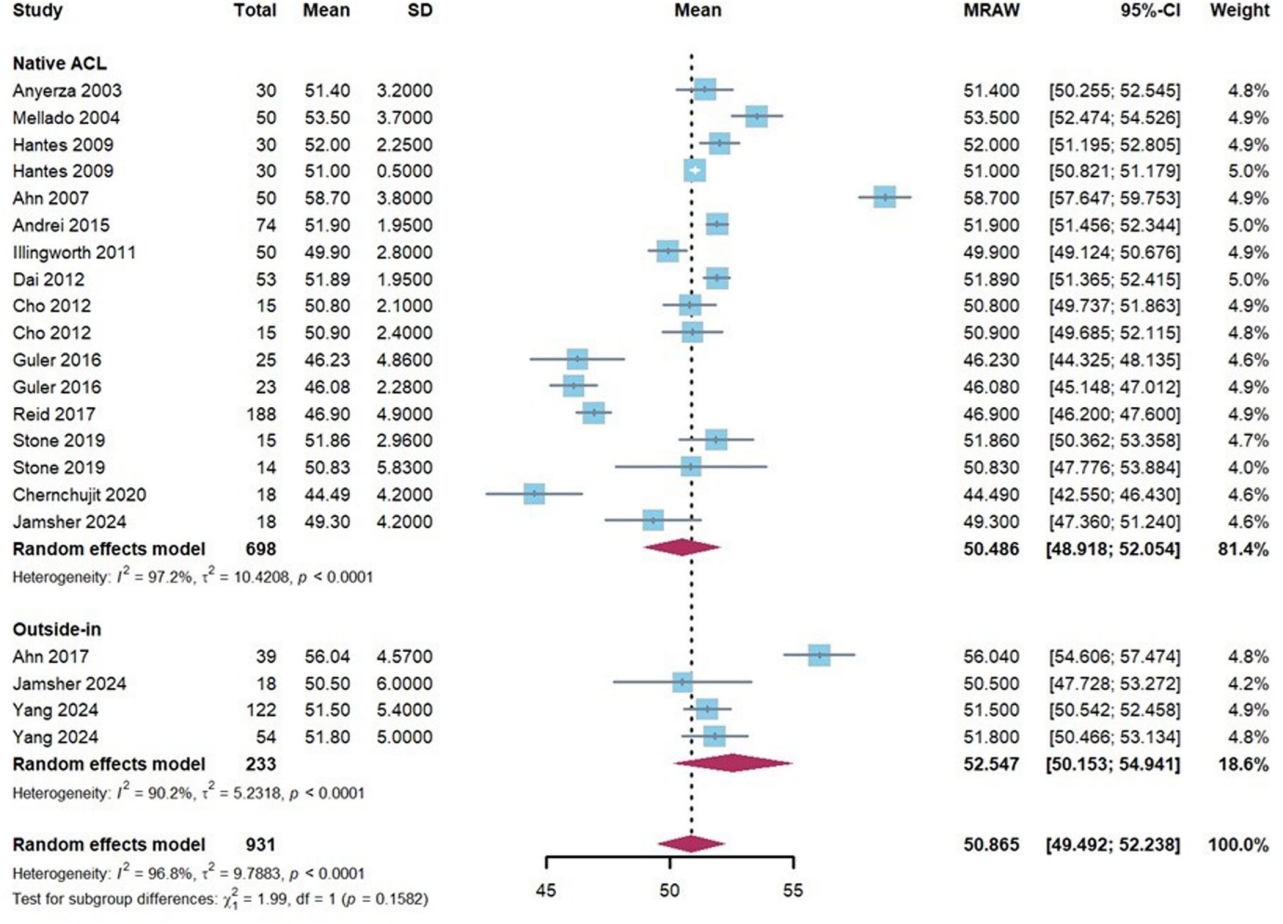
Sagittal inclination angles achieved with outside‐in/retrograde drilling technique compared to native values. CI, confidence interval; MRAW, mean raw value; SD, standard deviation.

Four studies evaluating outside‐in drilling techniques were included [2, 27, 56] (Figure 3). The pooled mean SIA for this subgroup was 52.5° (95% CI 50.2°–54.9°), with significant heterogeneity (*I* ^2^ ≈ 90%, *p* < 0.0001). SIAs achieved with outside‐in drilling closely approximated native ACL values, with overlapping CIs and no significant subgroup difference (*p* = 0.16).

### Anteromedial portal drilling–flexible reamers

**Figure 4:**
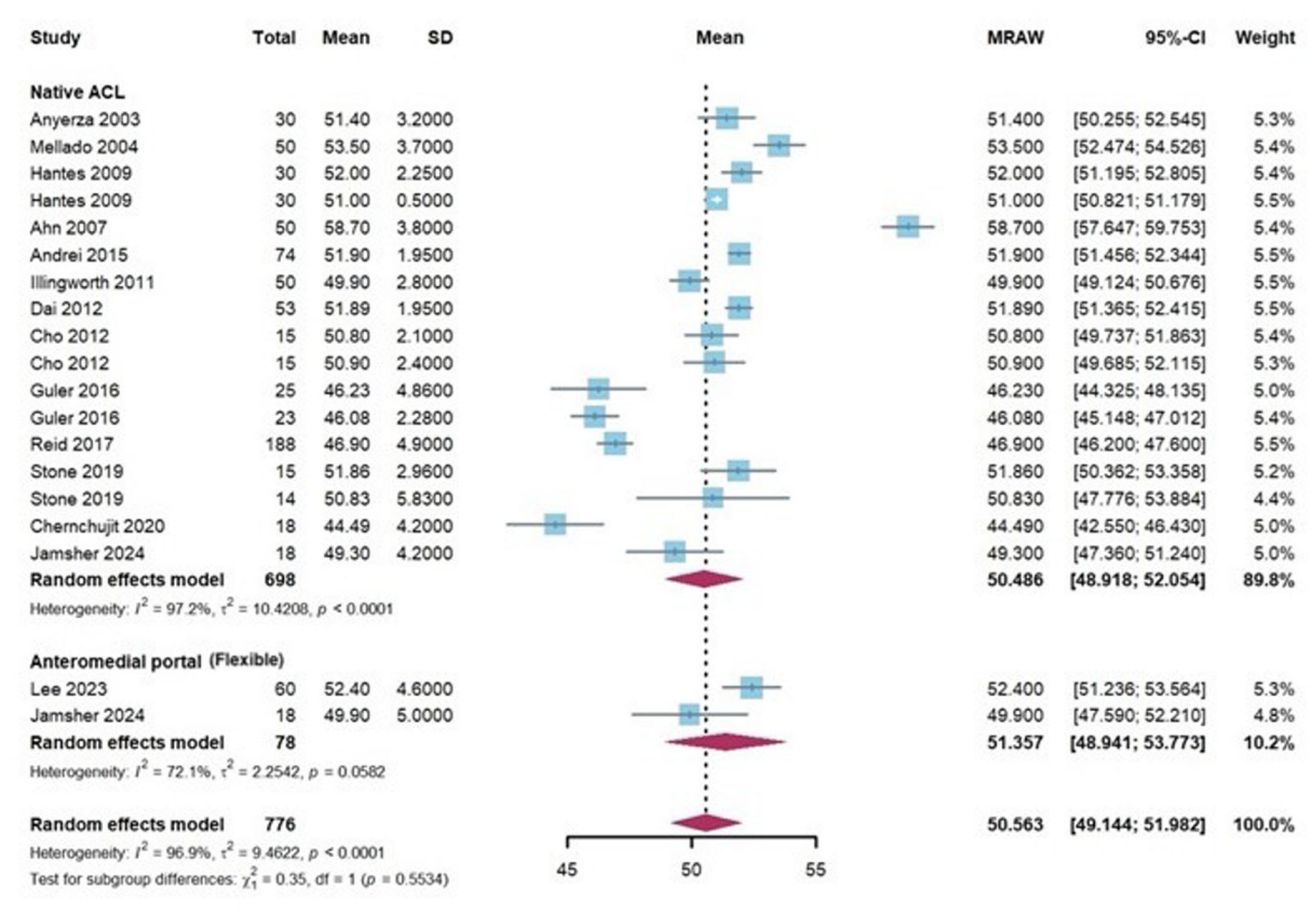
Sagittal inclination angles achieved with anteromedial portal drilling technique with flexible reamers compared to native values. CI, confidence interval; MRAW, mean raw value; SD, standard deviation.

Two studies evaluating anteromedial portal drilling techniques with flexible reamers were included (Figure 4). The pooled mean SIA for this subgroup was 51.4° (95% CI 48.9°–53.8°), with significant heterogeneity (*I* ^2 ^≈ 72%, *p* = 0.058). No statistically significant difference in SIA was observed between anteromedial portal reconstruction and native ACL values on subgroup testing (*p* = 0.55).

### Anteromedial portal drilling–rigid reamers

**Figure 5:**
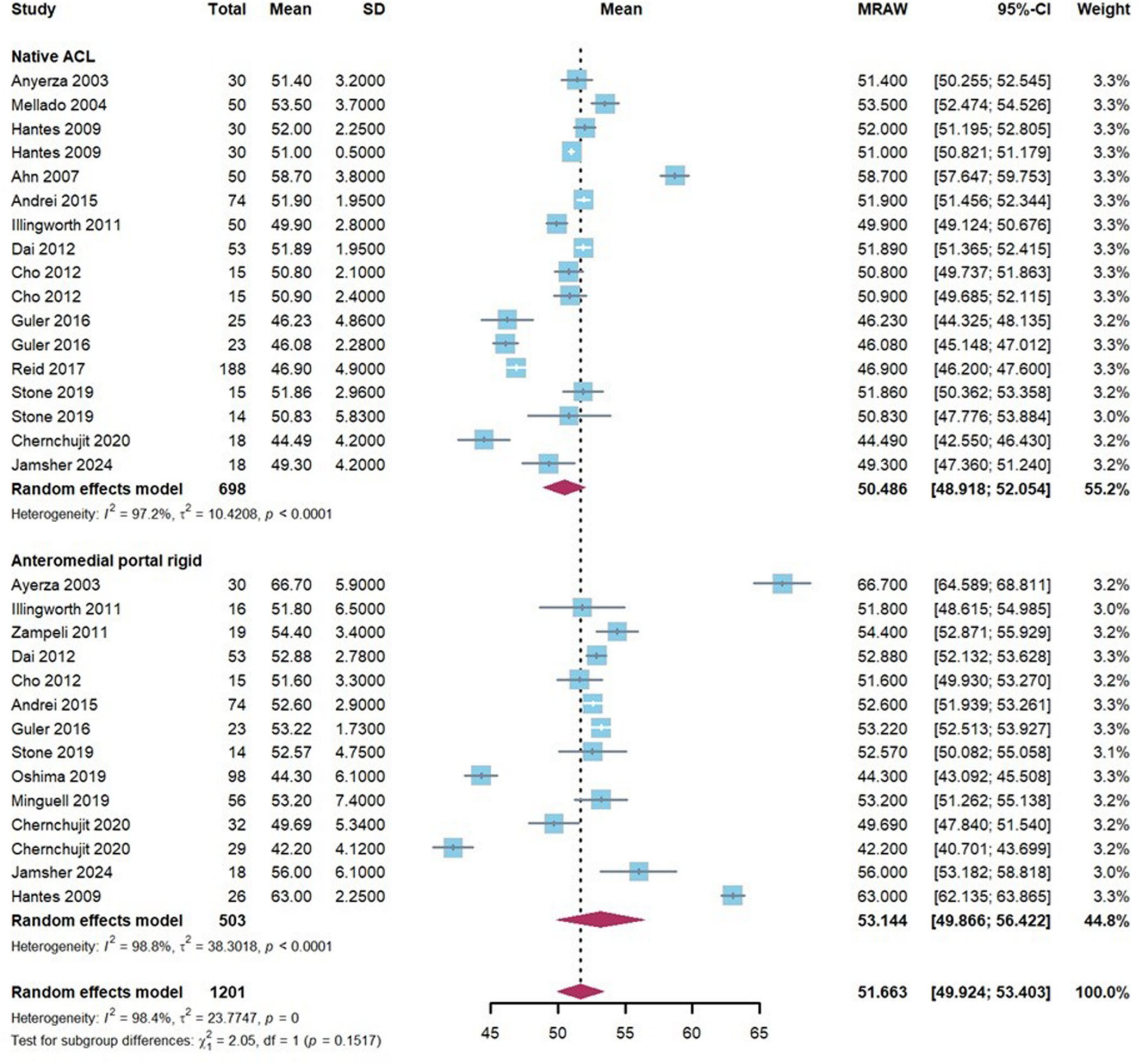
Sagittal inclination angles achieved with anteromedial portal drilling technique with rigid reamers compared to native values. CI, confidence interval; MRAW, mean raw value; SD, standard deviation.

Thirteen studies reported SIAs following anteromedial portal drilling with rigid reamers (Figure 5). The pooled mean SIA was 53.1° (95% CI 49.9°–56.4°), with significant heterogeneity (*I* ^2^ ≈ 99%, *p* < 0.0001). Subgroup comparison demonstrated no statistically significant difference relative to native ACL SIAs (*p* = 0.15).

### Transtibial drilling

**Figure 6:**
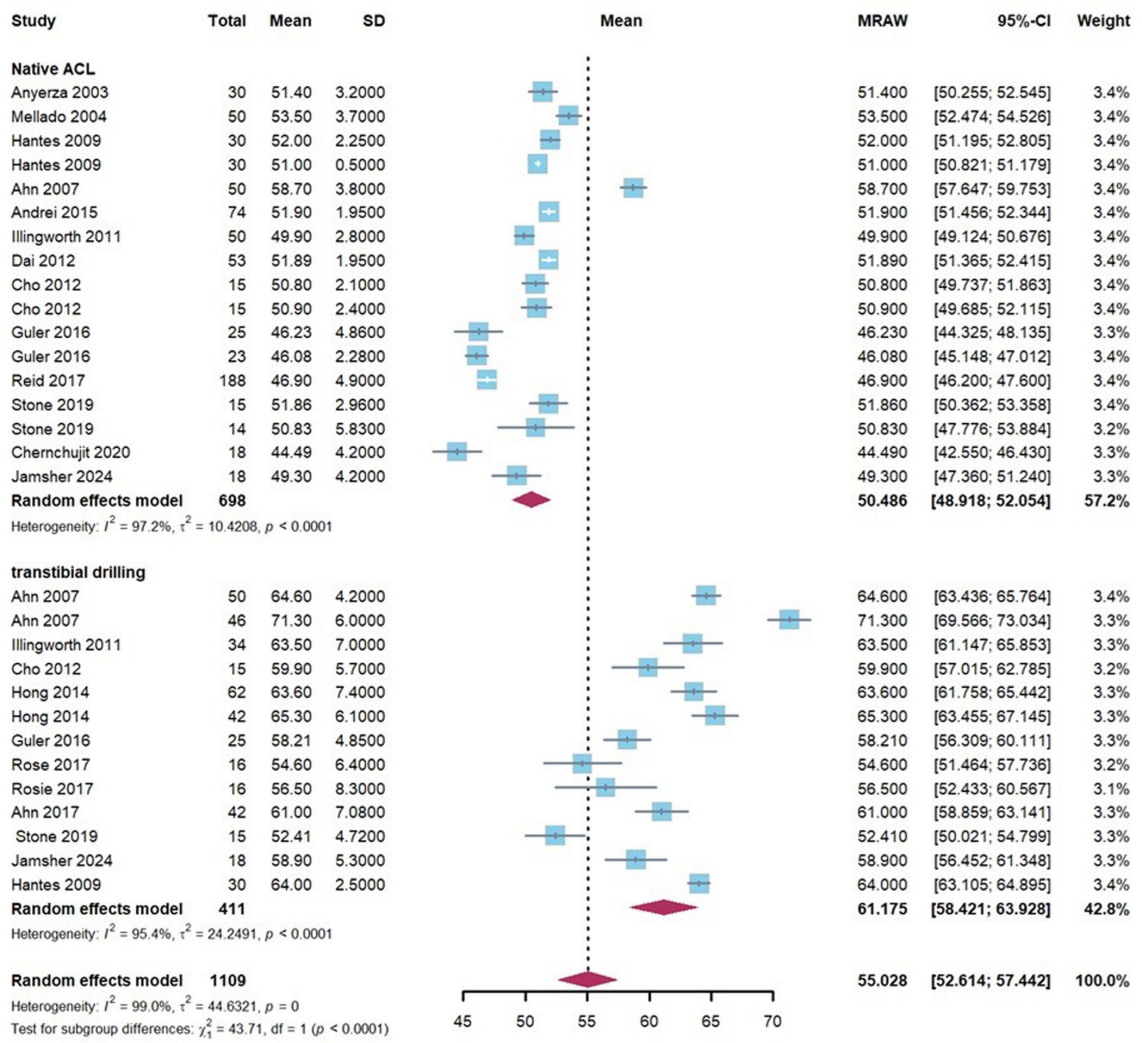
Sagittal inclination angles achieved with transtibial drilling technique compared to native values. CI, confidence interval; MRAW, mean raw value; SD, standard deviation.

Ten studies reported SIAs following transtibial ACL reconstruction (Figure 6). The pooled mean SIA for the transtibial subgroup was 61.2° (95% CI 58.4°–63.9°), with significant heterogeneity (*I* ^2 ^≈ 95%, *p* < 0.001). Subgroup analysis demonstrated a statistically significant difference between transtibial reconstruction and native ACL SIAs (*p* < 0.001), with transtibial techniques consistently producing higher sagittal inclination values.

### CIA

#### Native ACL

Across the six included studies, the pooled CIA of the native ACL was 67.5° (95% CI 63.3°–71.8°), with significant heterogeneity observed (*I* ^2^ ≈ 99%, *p* < 0.001). Individual study means demonstrated wide variability.

### Outside‐in/Retrograde drilling

Two studies reported CIAs following outside‐in femoral drilling (Supporting Information S1: Figure 1). The pooled CIA for this subgroup was 70.1° (95% CI 68.1°–72.0°), with minimal heterogeneity (*I* ^2^ = 0%). CIAs achieved with outside‐in drilling overlapped substantially with the native ACL range. Subgroup analysis did not demonstrate a statistically significant difference relative to native ACL CIAs (*p* = 0.30).

### Anteromedial portal drilling–flexible reamers

Two studies reporting CIAs following anteromedial portal drilling with flexible reamers were included (Supporting Information S1: Figure 2). The pooled mean CIA for this subgroup was 69.2° (95% CI 68.0°–70.4°), with minimal heterogeneity (*I* ^2^ = 0%). No statistically significant difference was observed compared with native ACL values (*p* = 0.46).

### Anteromedial portal drilling–rigid reamers

Six studies evaluating anteromedial portal drilling using rigid reamers were included (Supporting Information S1: Figure 3). The pooled mean CIA for this subgroup was 60.8° (95% CI 53.0°–68.7°), with significant heterogeneity (*I* ^2^ ≈ 99%, *p* < 0.001). Subgroup comparison demonstrated no statistically significant difference relative to native ACL CIAs (*p* = 0.14).

### Transtibial drilling

Eight studies reported CIAs following transtibial ACL reconstruction (Supporting Information S1: Figure 4). The pooled mean CIA for the transtibial subgroup was 70.6° (95% CI 67.2°–74.1°), with significant heterogeneity (*I* ^2^ ≈ 96%, *p* < 0.001). Subgroup analysis did not demonstrate a statistically significant difference relative to native ACL CIAs (*p* = 0.28).

### Summary of subgroup comparisons

SIA differed significantly by reconstruction technique, with transtibial drilling demonstrating consistently higher SIAs relative to native ACL anatomy. In contrast, CIAs overlapped substantially across reconstruction techniques, with no statistically significant differences observed relative to native ACL values.

## DISCUSSION

Pooled MRI‐based, tibial‐axis–referenced inclination angle measurements across native and reconstructed ACLs provide an anatomy‐referenced framework for comparison of reconstruction techniques at a population level. The synthesis defines a reproducible range of SIAs and CIAs for the native ACL and demonstrates that most contemporary femoral drilling approaches achieve graft orientations comparable to native anatomy, whereas transtibial reconstruction is associated with systematically higher SIAs. These findings address three persistent challenges in ACL reconstruction. First, postoperative MRI is the most commonly available, radiation‐free imaging modality for assessing graft placement, yet its interpretation has been limited by the absence of a pooled, anatomy‐referenced inclination benchmark. Second, surgeons increasingly select between transtibial, anteromedial portal and outside‐in techniques without quantitative evidence regarding the inclination angles each technique reliably reproduces. Third, deviation from native graft inclination has been associated with non‐anatomic tunnel position and adverse outcomes, making an anatomy‐referenced MRI standard clinically relevant.

Although individual study means varied, the clustering of native sagittal inclination values around 50° across diverse populations and imaging protocols supports the robustness of this estimate when measured using a tibial‐axis–based reference. In contrast, the pooled native CIA was higher, approximately 67° and demonstrated greater variability. This wider dispersion likely reflects both methodological heterogeneity and the less well‐defined biomechanical importance of coronal ACL orientation, consistent with the smaller number of studies and lower emphasis placed on coronal measurements in the literature. When considered in this context, commonly cited sagittal and coronal inclination thresholds proposed by the European Society of Sports Traumatology, Knee Surgery and Arthroscopy (ESSKA) guidelines [51] (<60° sagittal, <75° coronal) appear to align well with the pooled native ACL ranges identified in this analysis.

Addressing the second objective, comparison of reconstruction techniques relative to native ACL anatomy revealed a clear technique‐dependent pattern in the sagittal plane. Anteromedial portal drilling (using either rigid or flexible reamers) and outside‐in techniques achieved pooled SIAs that closely approximated the native ACL, with overlapping CIs and no statistically significant subgroup differences. These findings suggest that techniques permitting independent femoral tunnel positioning reliably reproduce native sagittal graft orientation on MRI. In contrast, transtibial reconstruction demonstrated a significantly higher pooled SIA, exceeding native ACL values by approximately 9°–10°, with subgroup testing confirming a statistically significant difference. This systematic elevation in sagittal inclination with transtibial drilling mirrors prior biomechanical and imaging studies linking higher sagittal angles to anterior femoral tunnel placement [17, 22, 25].

It is evident from the existing literature that SIA carries greater clinical and biomechanical relevance than CIA, a distinction reflected in the substantially larger number of studies evaluating sagittal graft orientation. Higher SIA values on MRI have been consistently associated with non‐anatomic graft placement secondary to anterior femoral tunnel malposition and with adverse biomechanical and clinical outcomes [17, 22, 38, 41, 60]. Conversely, excessively low sagittal inclination has also been implicated in suboptimal graft biology, with Li et al. demonstrating a strong negative association between SIA and graft signal–noise quotient at early follow‐up, suggesting impaired graft maturation [32, 43]. In contrast, evidence linking CIA to clinically meaningful outcomes remains limited [13, 34], and CIA appears less sensitive to variations in femoral drilling strategy. The wider 95% CIs observed for pooled CIA estimates likely reflect both genuine biological variability in coronal ACL orientation and methodological variability in coronal measurement technique, as the medial‐border tracing across sequential coronal slices is more operator‐dependent than the single‐plane sagittal measurement. The precision and clinical interpretability of CIA findings should therefore be considered with this in mind. The present meta‐analysis reinforces this distinction, demonstrating technique‐dependent differences in sagittal inclination while coronal inclination values largely overlapped across reconstruction techniques and the native ACL range. Interobserver agreement for inclination angle measurements was consistently high across the included studies that reported reliability, with intraclass correlation coefficients ranging from 0.89 to 0.96 for SIA and 0.85 to 0.94 for CIA measurements [16, 27, 30, 49], supporting the reproducibility of MRI‐based inclination assessment.

However, the present findings should not be interpreted to suggest that the use of anteromedial portal or retrograde drilling techniques guarantees achievement of anatomically accurate inclination angles. A central principle shared by these techniques is the surgeon's ability to arthroscopically identify the femoral ACL insertion footprint. This may be facilitated by various methods, including offset guides, direct visualization, calibrated rulers or fluoroscopy; however, none has been definitively shown to be superior. Ultimately, all techniques rely on the surgeon's judgement in positioning and accepting the trial guidewire prior to definitive tunnel or socket preparation.

It is well established that there is considerable anatomical variability in the location of the femoral ACL footprint [11, 12, 44]. Even experienced, fellowship‐trained surgeons demonstrate variability when asked to identify the femoral centre on three‐dimensional models [29], cadavers [14, 19] and in vivo arthroscopically [20]. Accordingly, the present meta‐analysis cannot conclusively determine superiority among independent femoral drilling techniques, beyond demonstrating that transtibial reconstruction is systematically associated with higher SIAs relative to native ACL values. Further, the established over‐the‐top technique [5, 35, 57, 59] lacks published data reporting SIA and CIA [39]; therefore, its ability to restore native graft orientation could not be directly compared with contemporary techniques in the present analysis. Theoretically, such techniques may exhibit reduced variability given the absence of femoral tunnel positioning; however, this remains to be formally evaluated.

An important methodological consideration is that the included studies did not, in general, report radiographic verification of femoral tunnel position (e.g., via the Bernard–Hertel grid or Forsythe quadrant method), nor were patients excluded on the basis of subsequently confirmed malposition. The pooled inclination angles reported here therefore represent each technique as practised in real‐world clinical cohorts, rather than the angles theoretically achievable under ideal tunnel positioning. Within‐technique heterogeneity may partly reflect this variation in tunnel‐positioning accuracy. Importantly, the systematic elevation in sagittal inclination observed with transtibial reconstruction occurred consistently across studies, surgeons and cohorts, most plausibly reflecting an inherent constraint of the transtibial technique rather than isolated malposition events.

A key limitation of this meta‐analysis is the substantial heterogeneity observed across analyses, likely reflecting differences in study populations, MRI acquisition protocols and timing of postoperative imaging. In subgroups with very high heterogeneity (*I* ^2 ^> 90%), the pooled estimate should be interpreted as an approximately unweighted average of study‐level means rather than a precision‐weighted summary, and between‐technique comparisons in these subgroups should be interpreted with corresponding caution. Nonetheless, by incorporating between‐study variance (*τ* ^2^) into the analysis, the random‐effects model produces estimates that are less reliant on the assumption of a common underlying effect, and the significant subgroup difference observed between the native ACL and the transtibial reconstruction in SIA persisted despite accounting for this heterogeneity, supporting the robustness of the principal finding. The outside‐in/retrograde and anteromedial portal–flexible subgroups included only two to three studies each, limiting the precision of pooled estimates for those techniques. CIA comparisons drew on fewer studies than SIA comparisons across all subgroups and should be interpreted with correspondingly lower confidence. The timing of postoperative MRI varied across studies and was inconsistently reported, which may affect graft inclination measurements due to graft remodelling and ligamentisation. In addition, SIAs and CIAs represent surrogate measures of graft orientation and do not capture the full three‐dimensional anatomy or in vivo behaviour of the ACL.

Several deliberate design choices represent important methodological strengths. Inclusion was restricted to MRI‐based, tibial‐axis–referenced measurements [1, 37, 51] performed on true sagittal imaging planes to ensure assessment of a consistent and biomechanically valid construct of ACL orientation. Studies using tibial plateau–based references, oblique imaging [47] or non‐standard measurement techniques [22, 48] were excluded to minimise construct heterogeneity and enhance comparability. In addition, focusing on predominantly skeletally mature patients [28] and single‐bundle reconstructions [17] using commonly employed graft types improves the clinical relevance and generalizability of the findings. Together, these methodological decisions allowed robust synthesis of native ACL inclination ranges and meaningful comparison across reconstruction techniques.

## CONCLUSION

The native ACL demonstrates a consistent SIA of approximately 50° on MRI when measured using a tibial‐axis reference, providing an anatomy‐referenced benchmark for the assessment of postoperative graft alignment. Anteromedial portal and outside‐in reconstruction techniques achieve sagittal and coronal graft orientations comparable to the native ACL, whereas transtibial reconstruction produces systematically higher SIAs. These findings support the use of standardized, tibial‐axis–based MRI measurements for objective comparison of ACL reconstruction techniques against native anatomy.

## AUTHOR CONTRIBUTIONS

Mok Ying Ren conceptualized and drafted the manuscript. Wang Chen and Francis Fong assisted in literature review and analysis. James Hui, Dave Lee, Wang Ming and Stefano Zaffagnini provided senior advice. All authors critically reviewed the manuscript and approved the final version.

## CONFLICT OF INTEREST STATEMENT

Stefano Zaffagnini has received fees as a paid presenter/speaker and paid consultant for DePuy, a Johnson & Johnson Company, and Smith & Nephew. He is a board or committee member of the European Society of Sports Traumatology, Knee Surgery and Arthroscopy (ESSKA) and the International Society of Arthroscopy, Knee Surgery, and Orthopaedic Sports Medicine (ISAKOS). He also serves on the editorial or governing board of the Journal of Experimental Orthopaedics (JEO). The remaining authors declare no conflict of interest.

## ETHICS STATEMENT

The authors have nothing to report.

## Supporting information


**Supplementary Figure S1.** Coronal inclination angles achieved with Outside‐in/Retrograde drilling technique compared to native values;
**Supplementary Figure S2.** Coronal inclination angles achieved with Anteromedial Portal drilling technique with flexible reamers compared to native values;
**Supplementary Figure S3.** Coronal inclination angles achieved with Anteromedial Portal drilling technique with rigid reamers compared to native values;
**Supplementary Figure S4.** Coronal inclination angles achieved with Transtibial drilling technique compared to native values;
**Supplementary Table 1.** Search terminology used;
**Supplementary Table 2.** Bias assessment and quality evaluation;
**Supplementary Table 3.** Newcastle–Ottawa Scale quality assessment;
**Supplementary Table 4.** Studies stratified according to surgical technique used.

## Data Availability

The data that support the findings of this study are available from the corresponding author upon reasonable request.
