# Supplementary material for: Transtibial ACL reconstruction produces higher sagittal inclination angles than the native ACL: A systematic review and meta‐analysis of MRI‐based measurements in 2047 knees
Source: J Exp Orthop. 2026 Jul 6;13(3):e70840. doi: 10.1002/jeo2.70840 (PMC13335086; doi:10.1002/jeo2.70840)
Supplement: Supplementary file 1 — Supplementary Figure S1. Coronal inclination angles achieved with Outside‐in/Retrograde drilling technique compared to native values; Supplementary Figure S2. Coronal inclination angles achieved with Anteromedial Portal drilling technique with flexible reamers compared to native values; Supplementary Figure S3. Coronal inclination angles achieved with Anteromedial Portal drilling technique with rigid reamers compared to native values; Supplementary Figure S4. Coronal inclination angles achieved with Transtibial drilling technique compared to native values; Supplementary Table 1. Search terminology used; Supplementary Table 2. Bias assessment and quality evaluation; Supplementary Table 3. Newcastle–Ottawa Scale quality assessment; Supplementary Table 4. Studies stratified according to surgical technique used. [file JEO2-13-e70840-s001.docx]

**Supplementary Figures**

***Figure S1. Coronal inclination angles achieved with Outside-in/ Retrograde drilling technique compared to native values***


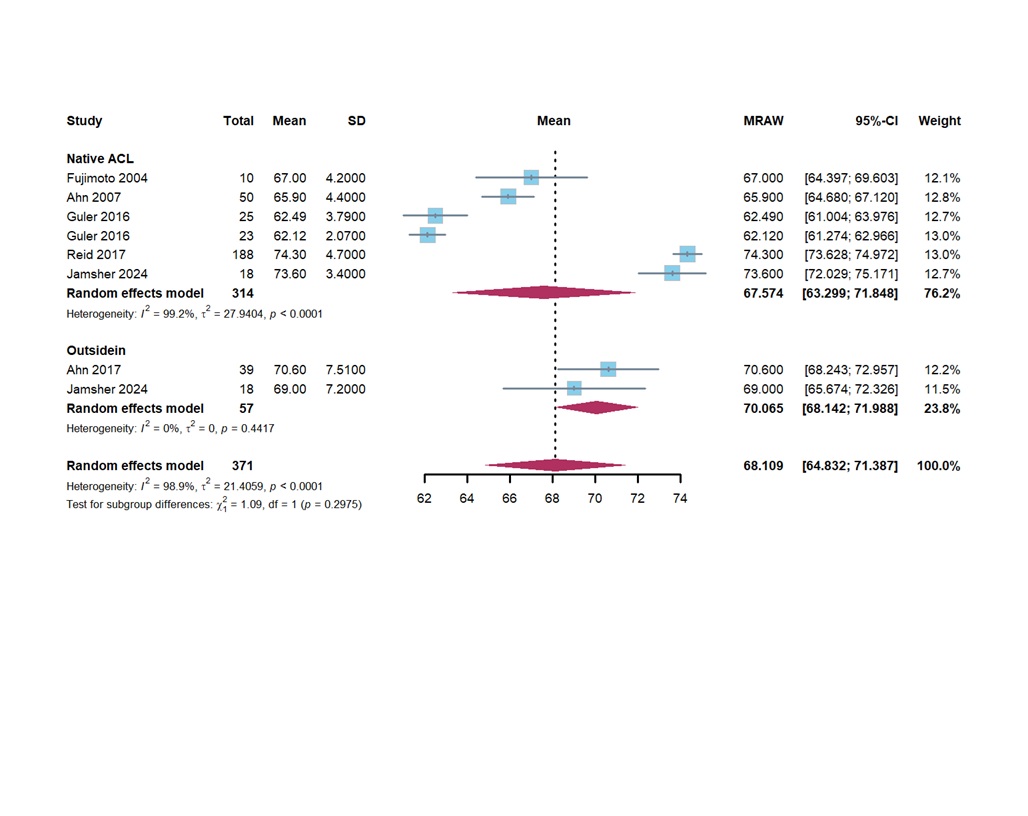


***Figure S2. Coronal inclination angles achieved with Anteromedial Portal drilling technique with flexible reamers compared to native values***


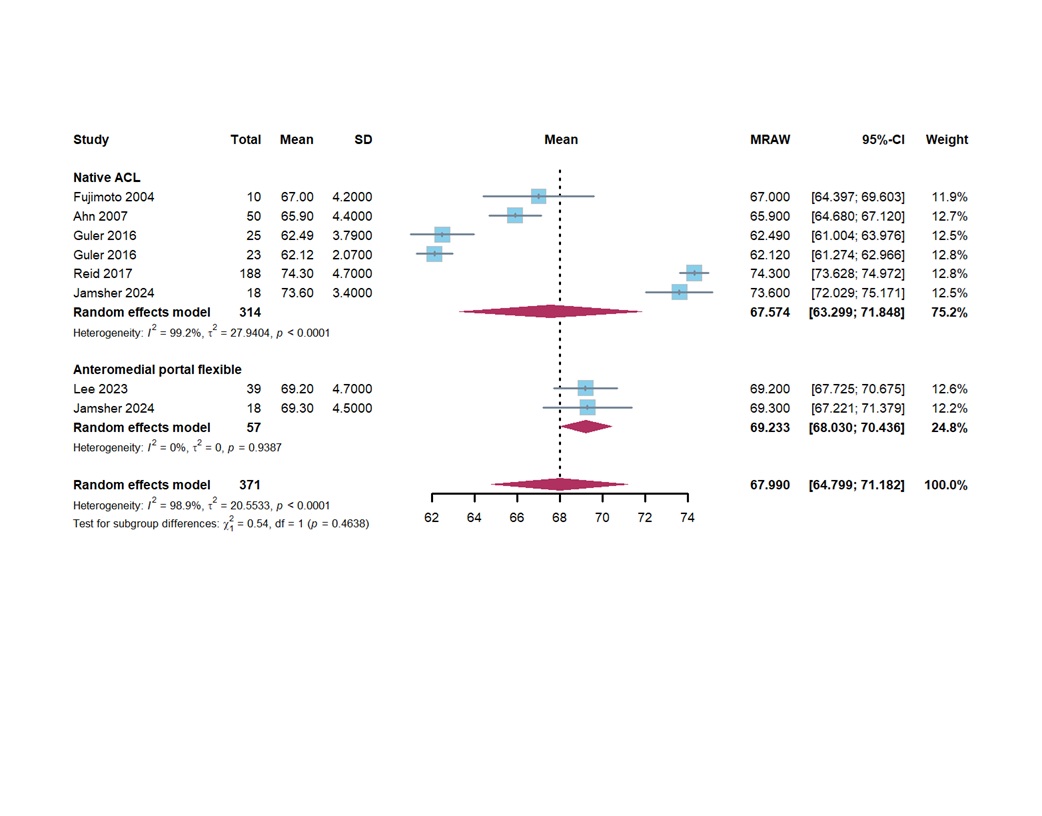


***Figure S3. Coronal inclination angles achieved with Anteromedial Portal drilling technique with rigid reamers compared to native values***


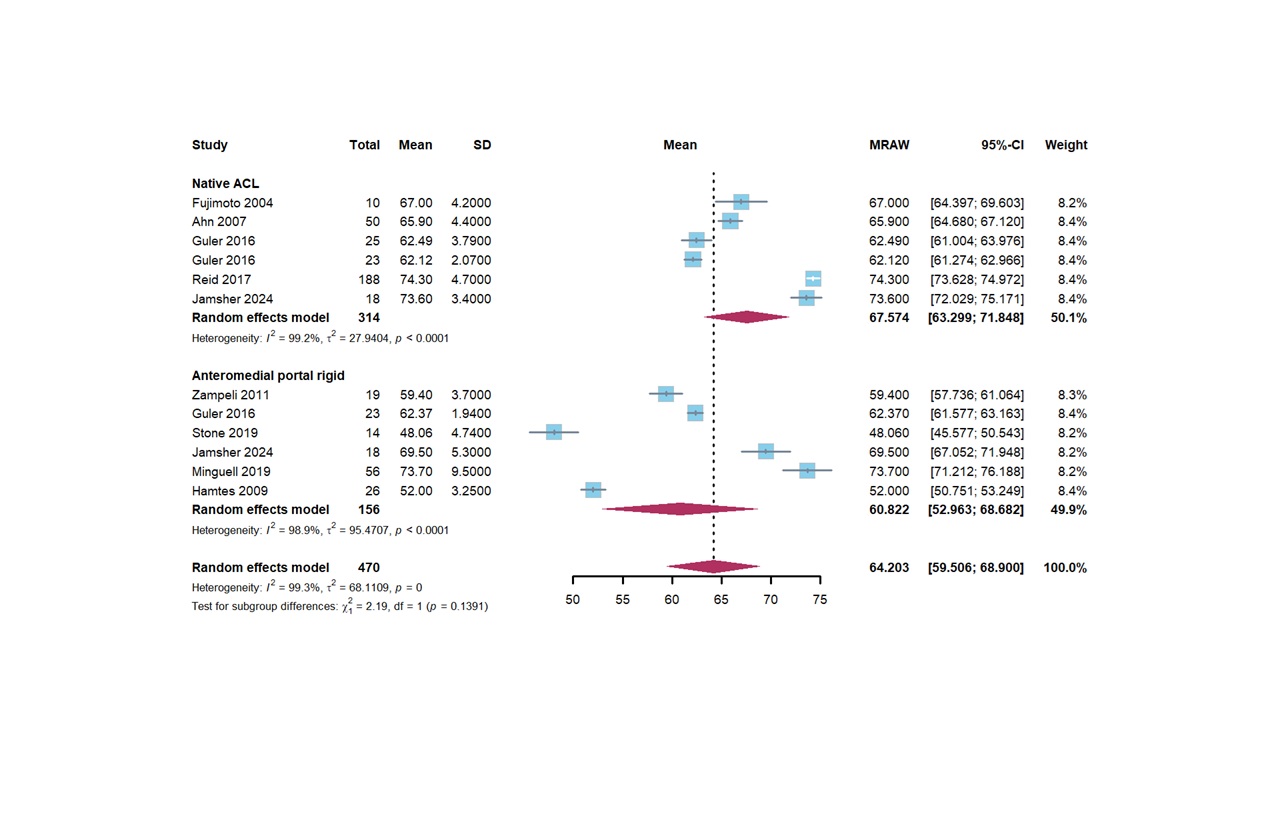


***Figure S4. Coronal inclination angles achieved with Transtibial drilling technique compared to native values***


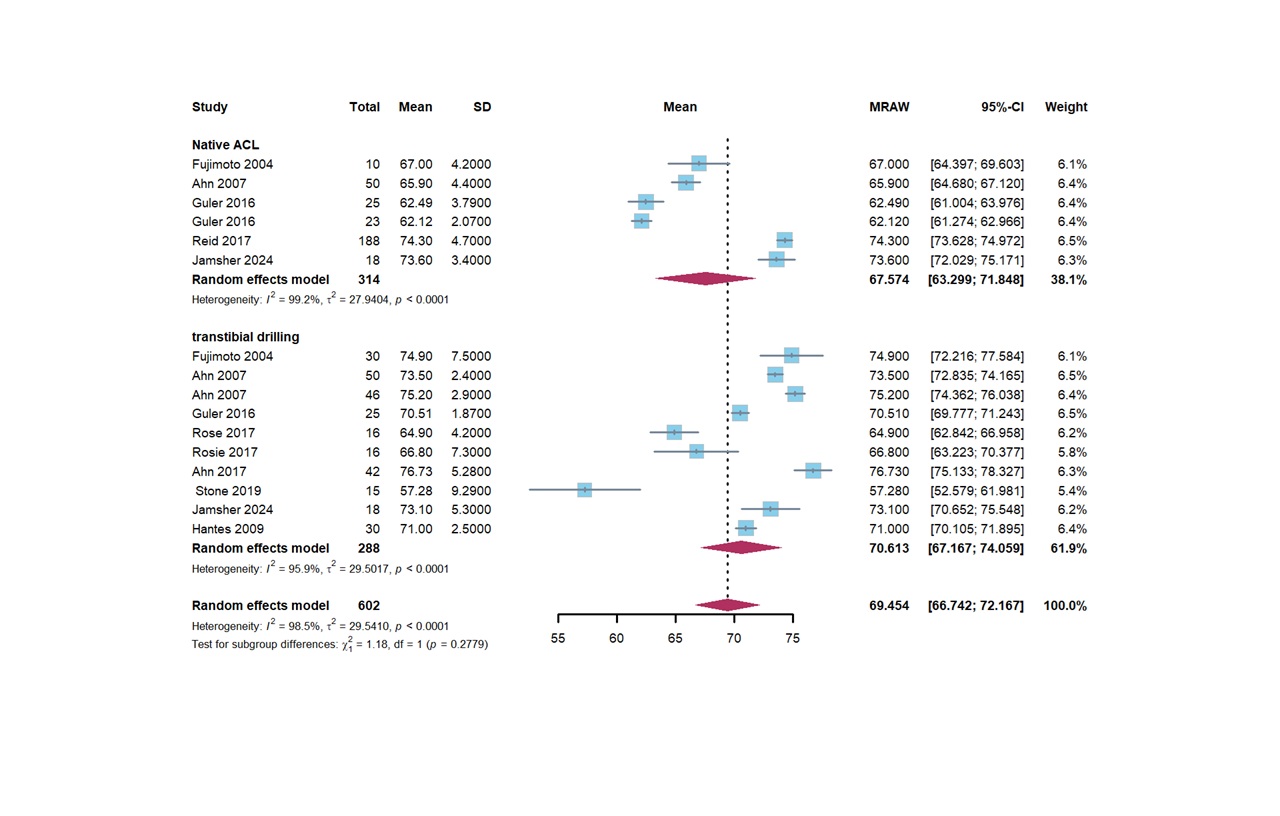


**Supplementary Table 1: Search Terminology Used**

| Search Term for Pubmed | 1. ( ("Anterior Cruciate Ligament Reconstruction"[Mesh] OR "ACL reconstruction" OR "ACL-R" OR "cruciate ligament reconstruction" OR "anterior cruciate ligament surgery") ) 2. ( ("femoral tunnel" OR "tibial tunnel" OR drilling OR technique* OR "outside-in" OR "retrograde drilling" OR "anteromedial portal" OR "transportal" OR "transtibial" OR "over-the-top" OR "all-inside" OR "anatomic" OR "non-anatomic") ) 3. ( ("graft inclination" OR "graft angle" OR "sagittal plane" OR "coronal plane" OR "three-dimensional orientation" OR obliquity OR “Sagittal inclination angle” OR “sagittal obliquity” OR “Sagittal ACL angle” OR “Sagittal graft angle” OR “Sagittal angle” OR “Sagittal inclination”)) 4. #1 AND #2 AND #3 |
| --- | --- |
| Search Term for Embase, Web of Science and Cochrane | 1. ('anterior cruciate ligament reconstruction'/exp OR 'anterior cruciate ligament reconstruction' OR 'acl reconstruction'/exp OR 'acl reconstruction' OR 'acl-r' OR 'cruciate ligament reconstruction' OR 'anterior cruciate ligament surgery'/exp OR 'anterior cruciate ligament surgery') 2. ('femoral tunnel'/exp OR 'femoral tunnel' OR 'tibial tunnel'/exp OR 'tibial tunnel' OR 'drilling'/exp OR drilling OR technique* OR 'outside-in' OR 'retrograde drilling'/exp OR 'retrograde drilling' OR 'anteromedial portal'/exp OR 'anteromedial portal' OR 'transportal' OR 'transtibial' OR 'over-the-top' OR 'all-inside' OR 'anatomic' OR 'non-anatomic') 3. (("graft inclination" OR "graft angle" OR "sagittal plane" OR "coronal plane" OR "three-dimensional orientation" OR obliquity OR “Sagittal inclination angle” OR “sagittal obliquity” OR “Sagittal ACL angle” OR “Sagittal graft angle” OR “Sagittal angle” OR “Sagittal inclination”)) 4. #1 AND #2 AND #3 |
| Search Term for Scopus | 1. (ALL(( 'femoral tunnel' / exp OR 'femoral tunnel' OR 'tibial tunnel' / exp OR 'tibial tunnel' OR 'drilling' / exp OR drilling OR technique* OR 'outside-in' OR 'retrograde drilling' / exp OR 'retrograde drilling' OR 'anteromedial portal' / exp OR 'anteromedial portal' OR 'transportal' OR 'transtibial' OR 'over-the-top' OR 'all-inside' OR 'anatomic' OR 'non-anatomic' ) ) ) 2. (ALL( ( 'anterior cruciate ligament reconstruction' / exp OR 'anterior cruciate ligament reconstruction' OR 'acl reconstruction' / exp OR 'acl reconstruction' OR 'acl-r' OR 'cruciate ligament reconstruction' OR 'anterior cruciate ligament surgery' / exp OR 'anterior cruciate ligament surgery' ) ) ) 3. #1 AND #2 |

**Supplementary Table 2: Bias assessment and quality evaluation**

| **Studies** | **Risk of Bias** |  |  |  |  |  |  |  |  |  |  |
| --- | --- | --- | --- | --- | --- | --- | --- | --- | --- | --- | --- |
|  | **1** | **2** | **3** | **4** | **5** | **6** | **7** | **8** | **9** | **10** | **Summary** |
| Ayerza et al 2003 [5] | LR | LR | HR | LR | LR | LR | LR | LR | NA | LR | LR |
| Mellado et al 2004 [37] | LR | LR | HR | LR | LR | LR | LR | LR | NA | LR | LR |
| Fujimoto et al 2004 [13] | LR | LR | HR | LR | LR | LR | LR | LR | NA | LR | LR |
| Hantes et al 2009 [18] | LR | LR | HR | LR | LR | LR | LR | LR | NA | LR | LR |
| Ahn et al 2007 [1] | LR | LR | HR | LR | LR | LR | LR | LR | NA | LR | LR |
| Ahn et al 2017 [2] | LR | LR | HR | LR | LR | LR | LR | LR | NA | LR | LR |
| Andrei et al 2015 [3] | LR | LR | HR | LR | LR | LR | LR | LR | NA | LR | LR |
| Illingworth et al 2011 [25] | LR | LR | HR | LR | LR | LR | LR | LR | NA | LR | LR |
| Dai et al 2012 [10] | LR | LR | HR | LR | LR | LR | LR | LR | NA | LR | LR |
| Cho et al 2012 [8] | LR | LR | HR | LR | LR | LR | LR | LR | NA | LR | LR |
| Guler et al 2016 [16] | LR | LR | HR | LR | LR | LR | LR | LR | NA | LR | LR |
| Reid et al 2017 [42] | LR | LR | HR | LR | LR | LR | LR | LR | NA | LR | LR |
| Stone et al 2019 [49] | LR | LR | HR | LR | LR | LR | LR | LR | NA | LR | LR |
| Chernchujit et al 2020 [7] | LR | LR | HR | LR | LR | LR | LR | LR | NA | LR | LR |
| Jamsher et al 2024 [27] | LR | LR | HR | LR | LR | LR | LR | LR | NA | LR | LR |
| Yang et al 2024 [56] | LR | LR | HR | LR | LR | LR | LR | LR | NA | LR | LR |
| Lee et al 2024 [30] | LR | LR | HR | LR | LR | LR | LR | LR | NA | LR | LR |
| Zampeli et al 2011 [60] | LR | LR | HR | LR | LR | LR | LR | LR | NA | LR | LR |
| Oshima et al 2019 [41] | LR | LR | HR | LR | LR | LR | LR | LR | NA | LR | LR |
| Minguell et al 2019 [38] | LR | LR | LR | LR | LR | LR | LR | LR | NA | LR | LR |
| Rose et al 2017 [43] | LR | LR | HR | LR | LR | LR | LR | LR | NA | LR | LR |

**LR: Low Risk; MR: Moderate Risk HR: High Risk; NA: Not Applicable**

**Risk of Bias Domains as described by Hoy et al:**

**External Validity**

1. Was the study’s target population a close representation of the national population in relation to relevant variables, e.g age, sex, occupation?

2. Was the sampling frame a true or close representation of the target population?

3. Was some form of random selection used to select the sample, OR, was a census undertaken?

4. Was the likelihood of non-response bias minimal?

**Internal Validity**

5. Were data collected directly from the subjects (as opposed to a proxy)?

6. Was an acceptable case definition used in the study?

7. Was the study instrument that measured the parameter of interest (e.g prevalence of low back pain) shown to have reliability and validity (if necessary)?

8. Was the same mode of data collection used for all subjects?

9. Was the length of the shortest prevalence period for the parameter of interest appropriate?

10. Were the numerator(s) and denominator(s) for the parameter of interest appropriate?

**Supplementary Table 3: Newcastle Ottawa Scale**

| Studies | Selection | | | | Comparability | Outcome | | | Total |
| --- | --- | --- | --- | --- | --- | --- | --- | --- | --- |
|  | Representativeness of exposed cohort | Selection of non-exposed cohort | Ascertainment of exposure | Demonstration that outcome of interest was not present at start of study | Comparability of cohorts based on basis of design or analysis | Assessments of outcomes | Was follow-up long enough for outcomes to occur | Adequacy of follow-up of cohorts (≥2 years) |  |
| Ayerza et al 2003 [5] | ◊ | ◊ | ◊ | ◊ | ◊ | ◊ | ◊ |  | 7* |
| Mellado et al 2004 [37] | ◊ | ◊ | ◊ | ◊ | ◊ | ◊ | ◊ |  | 7* |
| Fujimoto et al 2004 [13] | ◊ | ◊ | ◊ | ◊ | ◊◊ | ◊ | ◊ |  | 8* |
| Hantes et al 2009 [18] | ◊ | ◊ | ◊ | ◊ | ◊◊ | ◊ | ◊ |  | 8* |
| Ahn et al 2007 [1] | ◊ | ◊ | ◊ | ◊ | ◊◊ | ◊ | ◊ | ◊ | 9* |
| Ahn et al 2017 [2] | ◊ |  | ◊ | ◊ | ◊◊ | ◊ | ◊ |  | 7* |
| Andrei et al 2015 [3] | ◊ | ◊ | ◊ | ◊ | ◊ | ◊ | ◊ |  | 7* |
| Illingworth et al 2011 [25] | ◊ | ◊ | ◊ | ◊ | ◊ | ◊ | ◊ |  | 7* |
| Dai et al 2012 [10] | ◊ | ◊ | ◊ | ◊ | ◊ | ◊ | ◊ |  | 7* |
| Cho et al 2012 [8] | ◊ | ◊ | ◊ | ◊ | ◊ | ◊ | ◊ |  | 7* |
| Guler et al 2016 [16] | ◊ | ◊ | ◊ | ◊ | ◊◊ | ◊ | ◊ |  | 8* |
| Reid et al 2017 [42] |  | ◊ | ◊ | ◊ | ◊◊ | ◊ | ◊ | ◊ | 8* |
| Stone et al 2019 [49] |  | ◊ | ◊ | ◊ | ◊◊ | ◊ | ◊ |  | 7* |
| Chernchujit et al 2020 [7] | ◊ | ◊ | ◊ | ◊ | ◊◊ | ◊ | ◊ |  | 8* |
| Jamsher et al 2024 [27] | ◊ | ◊ | ◊ | ◊ | ◊◊ | ◊ | ◊ |  | 8* |
| Yang et al 2024 [56] | ◊ |  | ◊ | ◊ | ◊◊ | ◊ | ◊ | ◊ | 8* |
| Lee et al 2024 [30] | ◊ | ◊ | ◊ | ◊ | ◊ | ◊ | ◊ |  | 7* |
| Zampeli et al 2011 [60] | ◊ | ◊ | ◊ | ◊ | ◊◊ | ◊ | ◊ |  | 8* |
| Oshima et al 2019 [41] | ◊ |  | ◊ | ◊ | ◊◊ | ◊ | ◊ | ◊ | 8* |
| Minguell et al 2019 [38] | ◊ |  | ◊ | ◊ | ◊◊ | ◊ | ◊ | ◊ | 8* |
| Rose et al 2017 [43] | ◊ |  | ◊ | ◊ | ◊◊ | ◊ | ◊ |  | 7* |

Supplementary Table 4: Studies Stratified According to Surgical Technique Used

| Study | Graft | Country | Study Design | No. of Knees | Age | Sex | SIA (°) | CIA (°) |
| --- | --- | --- | --- | --- | --- | --- | --- | --- |
| Studies reporting Native ACL values | | | | | | | | |
| Ayerza et al 2003 [5] | NA | Argentina | RCS | 30 | 30.1 ± 8.6 | NA | 51.4 ± 3.2 | NA |
| Mellado et al 2004 [37] | NA | Spain | RCS | 50 | 41.5 ± 13.1 | 31M/ 19F | 53.5 ± 3.7 | NA |
| Fujimoto et al 2004 [13] | NA | Japan | PCS | 10 | NA | NA | NA | 67.0 ± 4.2 |
| Hantes et al 2009 [18] | NA | Greece | RCS | 30 | 25.6 | 28M/ 2F | 52 ± 2.3 | NA |
| Hantes et al 2009 [18] | NA | Greece | RCS | 30 | 27.2 | 26M/ 4F | 51 ± 0.5 | NA |
| Ahn et al 2007 [1] | NA | Korea | RCS | 50 | 28.3 ± 9.9 | 39M/ 11F | 58.7 ± 3.8 | 65.9 ± 4.4 |
| Andrei et al 2015 [3] | NA | Romania | RCS | 74 | NA | NA | 51.9 ± 2.0 | NA |
| Illingworth et al 2011[25] | NA | USA | RCS | 50 | NA | NA | 49.9 ± 2.8 | NA |
| Dai et al 2012 [10] | NA | China | RCS | 53 | NA | NA | 51.9 ± 2.0 | NA |
| Cho et al 2012 [8] | NA | Korea | RCS | 15 | NA | NA | 50.8 ± 2.1 | NA |
| Cho et al 2012 [8] | NA | Korea | RCS | 15 | NA | NA | 50.9 ± 2.4 | NA |
| Guler et al 2016 [16] | NA | Turkey | RCS | 25 | 31.4 ± 4.1 | 25M / 0F | 46.2 ±4.9 | 62.5 ±3.8 |
| Guler et al 2016 [16] | NA | Turkey | RCS | 23 | 30.9 ± 3.7 | 22M/ 1F | 46.1 ±2.3 | 62.1 ±2.1 |
| Reid et al 2017 [42] | NA | USA | RCS | 188 | 152 Mature/ 36 Immature | 98M/ 90F | 46.9 ± 4.9 | 74.3 ± 4.7 |
| Stone et al 2019 [49] | NA | USA | PCS | 15 | 23.8 ± 3.3 | 11M 4F | 51.9 ±3.0 | NA |
| Stone et al 2019 [49] | NA | USA | PCS | 14 | 24.3 ± 3.6 | 11M 3F | 50.8 ±5.8 | NA |
| Chernchujit et al 2020 [7] | NA | India | RCS | 51 | 41.5 ± 12.8 | NA | 44.5 ± 4.7 | NA |
| Jamsher et al 2024 [27] | NA | Switzerland | PCS | 18 | 38.0 ± 11.4 | 13M/ 5F | 49.3 ± 4.2 | 73.6 ± 3.4 |
| Studies that utilized outside-in/retrograde drilling | | | | | | | | |
| Ahn et al 2017 [2] | HS | Korea | RCS | 39 | 31.4 ± 11.1 | 33M/ 6F | 56.0 ±4.6 | 70.6 ±7.5 |
| Jamsher et al 2024 [27] | HS | Switzerland | PCS | 18 | 29.4 ± 8.3 | 14M/ 4F | 50.5 ± 6.0 | 69.0 ± 7.2 |
| Yang et al 2024 [56] | HS | Korea | RCS | 122 | 28.7 ± 12 | 100M/ 22F | 51.5 ± 5.4 | NA |
| Yang et al 2024 [56] | HS | Korea | RCS | 54 | 26.7 ± 10.6 | 44M / 10F | 51.8 ± 5.0 | NA |
| Studies that utilized anteromedial portal drilling (Flexible) | | | | | | | | |
| Lee et al 2023 [30] | HS/ TAA | Korea | RCS | 60 | 28.4 ± 9.9 | 37M/ 23F | 52.4 ± 4.6 | 69.2 ± 4.7 |
| Jamsher et al 2024 [27] | HS | Switzerland | PCS | 18 | 33.4 ± 12.6 | 13M/ 5F | 49.9 ± 5.0 | 69.3 ± 4.5 |
| Studies that utilized anteromedial portal drilling (Rigid) | | | | | | | | |
| Ayerza et al 2003 [5] | BPTB | Argentina | RCS | 30 | 32.3 ± 8.7 | NA | 66.7 ± 5.9 | NA |
| Illingworth et al 2011 [25] | NA | USA | RCS | 16 | NA | NA | 51.8 ± 6.5 | NA |
| Zampeli et al 2011 [60] | BPTB | Greece | RCS | 19 | 29 ± 7.4 | 19M | 54.4 ± 3.4 | 59.4 ± 3.7 |
| Dai et al 2012 [10] | NA | China | RCS | 53 | NA | NA | 52.88 ± 2.78 | NA |
| Cho et al 2012 [8] | QT/ ATA | Korea | RCS | 15 | NA | NA | 51.6 ± 3.3 | NA |
| Andrei et al 2015 [3] | HS | Romania | RCS | 74 | NA | NA | 52.6 ± 2.9 | NA |
| Guler et al 2016 [16] | HS | Turkey | RCS | 23 | 30.9 ± 3.7 | 22M/ 1F | 53.2 ±1.7 | 62.4 ±1.9 |
| Stone et al 2019 [49] | BPTB | USA | PCS | 14 | 24.3 ± 3.6 | 11M/ 3F | 52.6 ±4.8 | 48.1 ± 4.7 |
| Oshima 2019 [41] | HS | Japan | RCS | 98 | 32.5 ± 11.7 | 58M/ 40F | 44.3 ± 6.1 | NA |
| Minguell et al 2019 [38] | HS | Spain | RCT | 56 | 31 ± 9.7 | 36M/ 19F | 53.2 ± 7.4 | 73.7 ± 9.5 |
| Chernchujit et al 2020 [7] | HS | India | RCS | 32 | 32.1 ± 9.4 | NA | 49.7 ± 5.3 | NA |
| Chernchujit et al 2020 [7] | HS | India | RCS | 29 | 34.8 ± 9.9 | NA | 42.2 ± 4.1 | NA |
| Jamsher et al 2024 [27] | HS | Switzerland | PCS | 18 | 27.5 ± 7.2 | 17M/ 1F | 56.0 ± 6.1 | 69.5 ± 5.3 |
| Hantes et al 2009 [18] | HS | Greece | RCS | 26 | 27.2 | 26M/4F | 63 ± 2.25 | 52 ± 3.25 |
| Studies that utilized Transtibial Drilling | | | | | | | | |
| Fujimoto et al 2004 [13] | HS | Japan | PCS | 30 | 26.3 (15 – 53) | 18M/ 12F | NA | 74.9 ± 7.5 |
| Ahn et al 2007 [1] | HS | Korea | RCS | 50 | 31.7 ± 9.9 | 40M/ 10F | 64.6 ± 4.2 | 73.5 ± 2.4 |
| Ahn et al 2007 [1] | BPTB | Korea | RCS | 46 | 29.4 ± 8.4 | 35M/ 11F | 71.3 ± 6.0 | 75.2 ± 2.9 |
| Illingworth et al 2011 [25] | NA | USA | RCS | 34 | NA | NA | 63.5 ± 7.0 | NA |
| Cho et al 2012 [8] | QT/ ATA | Korea | RCS | 15 | NA | NA | 59.9 ± 5.7 | NA |
| Hong Li et al 2014 [31] | TAA | China | RCS | 62 | 29.9 ± 6.9 | 62M / 0F | 63.6 ± 7.4 | NA |
| Hong Li et al 2014 [31] | HS | China | RCS | 42 | 29.0 ± 5.5 | 42M / 0F | 65.3 ± 6.1 | NA |
| Guler et al 2016 [16] | HS | Turkey | RCS | 25 | 31.4 ± 4.1 | 25M / 0F | 58.2 ±4.9 | 70.5 ±1.9 |
| Rose et al 2017 [43] | TAA | USA | PCS | 16 | 45.0 ± 12.5 | 7M/ 9F | 54.6 ± 6.4 | 64.9 ±4.2 |
| Rose et al 2017 [43] | HS | USA | PCS | 16 | 37.0 ± 12.0 | 11M/ 5F | 56.5 ± 8.3 | 66.8 ± 7.3 |
| Ahn et al 2017 [2] | HS | Korea | RCS | 42 | 29.7 ± 9.4 | 35M/ 7F | 61.0 ± 7.1 | 76.7 ±5.3 |
| Minguell et al 2019 [38] | HS | Spain | RCT | 51 | 29.8 ± 8.8 | 38M/ 13F | 59.5 ± 5.1 | 79.1 ± 6.1 |
| Stone et al 2019 [49] | BPTB | USA | PCS | 15 | 23.8 ± 3.3 | 11M 4F | 52.4 ± 4.7 | 57.3 ±9.3 |
| Jamsher et al 2024 [27] | HS | Switzerland | PCS | 18 | 32.9 ± 9.3 | 14M/ 4F | 58.9 ± 5.3 | 73.1 ± 5.3 |
| Hantes et al 2009 [18] | HS | Greece | RCS | 30 | 25.6 | 28M/2F | 64 ± 2.5 | 71 ± 2.5 |

*RCT: Randomised controlled Trial; RCS: Retrospective Cohort Study; PCS: Prospective Cohort Study
